# Minor Physical Anomalies in Patients with Schizophrenia, Unaffected First-Degree Relatives, and Healthy Controls: A Meta-Analysis

**DOI:** 10.1371/journal.pone.0024129

**Published:** 2011-09-08

**Authors:** Ting Xu, Raymond C. K. Chan, Michael T. Compton

**Affiliations:** 1 Neuropsychology and Applied Cognitive Neuroscience Laboratory, Key Laboratory of Mental Health, Institute of Psychology, Chinese Academy of Sciences, Beijing, China; 2 Graduate School, Chinese Academy of Sciences, Beijing, China; 3 Emory University School of Medicine, Department of Psychiatry and Behavioral Sciences, Atlanta, Georgia, United States of America; Chiba University Center for Forensic Mental Health, Japan

## Abstract

**Background:**

Minor physical anomalies (MPAs) have been found to be more prevalent in schizophrenia than control participants in numerous studies and may index a potential endophenotype for schizophrenia.

**Aim:**

To quantitatively define the magnitude of the difference in total MPA scores between patients with schizophrenia and healthy controls; to determine the degree of manifestation in unaffected first-degree relatives compared to patients and controls; and to investigate the degree of sensitivity among individual MPA items.

**Methods:**

A systematic search was conducted on the literature pertaining to MPAs in patients with schizophrenia and unaffected relatives. Effect sizes (Cohen's *d* and odds ratios) and corresponding confidence intervals were combined using the Comprehensive Meta-Analysis software package.

**Results:**

A large difference was found when examining 14 studies comprising 1207 patients with schizophrenia and 1007 healthy controls (*d* = 0.95, 95% CI = 0.63, 1.27). Six studies involving relatives of individuals with schizophrenia showed a medium effect size (*d* = 0.45, 95% CI = 0.29,0.62) between patients and relatives, but a small and non-significant effect size (*d* = 0.32, 95% CI = −0.08, 0.73) between relatives and controls. The majority of MPAs items showed significant odds ratios (1.26–9.86) in comparing patients and controls.

**Conclusions:**

The findings indicate that medium effect size of MPAs have been demonstrated in patients with schizophrenia as compared to healthy controls, and to a lesser extent in unaffected relatives. These findings are consistent with the idea that MPAs may represent a putative endophenotype for schizophrenia. However, more research including first-degree family members is warranted.

## Introduction

The *endophenotype* construct represents a promising approach to facilitating investigations of schizophrenia, a disorder that is now thought to be influenced by multiple genes as well as environmental factors. Gottesman and Shields described an endophenotype as an internal, intermediate phenotype (i.e., not obvious to the unaided eye) that fills the gap between genes and diseases [1]. Endophenotypes should be: (1) associated with the illness in the population, (2) heritable, (3) state-independent, (4) found in unaffected family members at a higher rate than in the general population, and (5) shown to co-segregate with the illness within families[2]. Multiple potential endophenotypes, such as specific cognitive deficits and neurological soft signs, have been examined in patients, relatives, and healthy controls [1], [3].

Minor physical anomalies (MPAs) are suggested as an endophenotype on account of the findings that MPAs present more in patients than healthy controls and are state-independent [4]. Weinberg and colleagues (2007) reviewed the studies on MPAs in schizophrenia and pooled effect sizes calculated from 11 studies by meta-analysis. The results revealed a high degree of difference (*d* = 1.13) between the patients with schizophrenia and healthy controls [5]. In addition, several studies have investigated the prevalence of MPAs in unaffected relatives of patients with schizophrenia, though consistency across the research literature has been limited. Therefore, we performed the present meta-analysis to: (1) update the literature about MPAs in patients with schizophrenia and control comparisons, (2) quantify the magnitude of the mean difference between relatives and patients with schizophrenia, and (3) determine the magnitude of the difference between relatives of patients with schizophrenia and healthy controls. As a secondary aim, we examined individual MPA items to determine which specific MPAs manifest more commonly in schizophrenia than in healthy controls across studies.

## Methods

### Literature Search

Relevant articles, published from 1968 through July 31^st^ 2011, were identified using an extensive literature search of Elsevier Science, Blackwell, Springer, PsycINFO, and Medline. The keywords were: “minor physical anomalies,” “MPA,” “morphologic signs,” “dysmorphic,” and “schizophrenia.” These search procedures yielded 86 potential articles for review.

#### (a) Inclusion criteria

**Figure 1:**
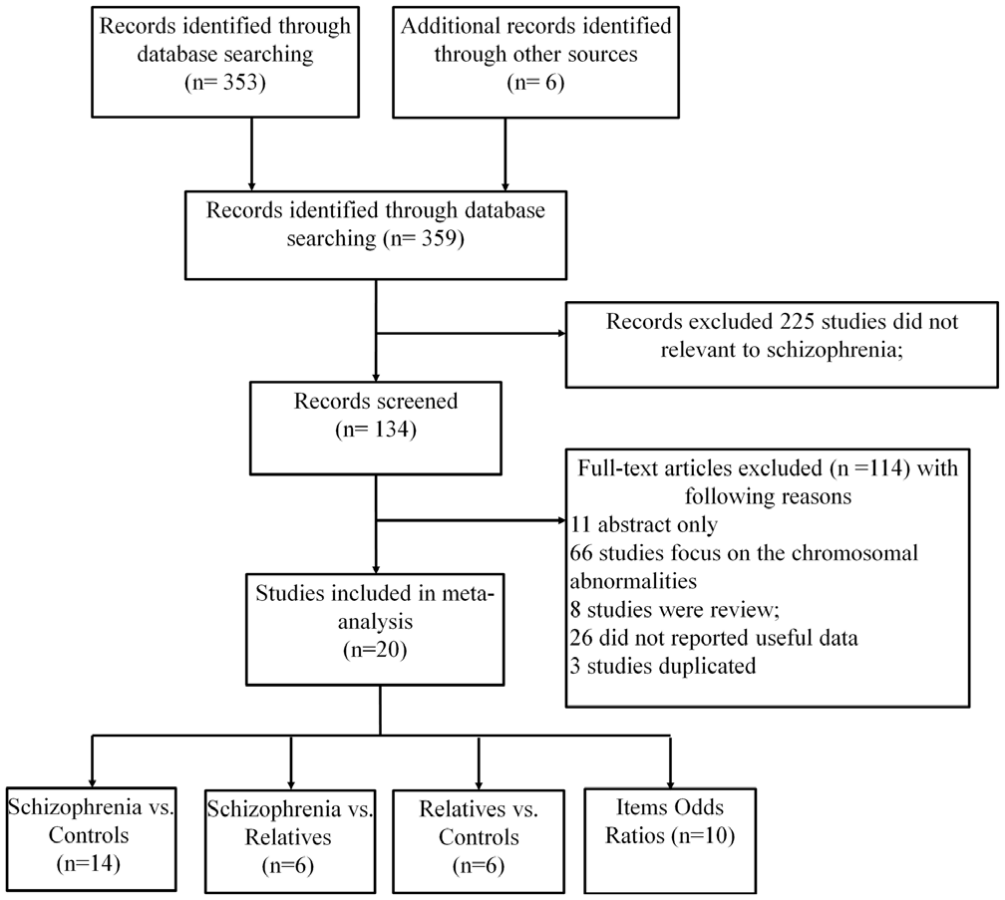
The flow chart for the inclusion and exclusion of studies for the current meta-analysis.

Studies selected for meta-analytic review met the following criteria: (1) diagnoses were based on diagnostic criteria for schizophrenia as operationalized in DSM-III, DSM-III-R, DSM-IV, ICD-9, or ICD-10; (2) a standardized scale was used to assess MPAs (e.g., the widely used Waldrop Scale [6]; (3) a healthy control group or a sample of first-degree relatives of patients with schizophrenia was included; and (4) MPA scores of at least two of the three groups (patients, relatives, and controls) were available as means and standard deviations (SDs), or an effect size was calculable via *t* or *F* test values. Based on the pool of available articles, using these inclusion criteria resulted in 19 eligible studies (Figure 1).

#### (b) Exclusion criteria

To ensure that more than one study included in the meta-analysis did not involve the same group of subjects, for studies in which an overlap of sample or data was identified, the one with the largest sample size was chosen. Several studies from the same research groups had apparently overlapping samples to varying degrees [7], [8], [9], [10], [11], [12], [13], [14]. Four studies were excluded after this procedure; thus, 14 studies remained that included comparisons between patients and controls, and six studies had data on first-degree relatives.

### Statistical Analyses

Effect sizes (Cohen's *d*) indexing the difference between groups were calculated by taking the difference in means between two groups, divided by the pooled standard deviation. Specifically, for schizophrenia versus healthy controls, the difference in means between two groups was the mean of the schizophrenia sample minus the mean of the healthy controls. Similarly, for schizophrenia versus relatives and relatives versus healthy controls, the difference in means were the mean of the schizophrenia group minus the mean of relatives and the mean of the relatives minus the mean of controls, respectively. The 95% confidence interval (CI) was estimated to determine whether effect sizes were statistically significant. Since the individual effect size distributions may be heterogeneous across studies, the Q statistic was computed. A significant Q statistic indicates heterogeneity of the individual study effect sizes [15]. Based on the Q statistic, moderator analyses were carried out to determine whether the number of MPA scale items, sex ratio among patients, and sex ratio among controls led to the heterogeneous results.

Meta-analysis has the potential to be affected by publication bias. The most possible publication bias is the lack of published studies yielding non-significant results [16]. The Orwin's fail-safe number estimates the number of non-significant, unpublished studies that would overturne an overall statistically significant observed effect to some specified and negligible level [17], [18]. We set the negligible level at 0.2, which indicates a weak and usually non-significant effect size [19]. In addition, we set 0.1 as the mean effect size of the hypothetical, additional “missing” studies.

Regarding the second aim, odds ratios (ORs) were calculated for each specific MPA item from studies that reported the percentage or the number represented in schizophrenia and healthy controls. Then, combined ORs with 95% confidence intervals and heterogeneity statistics were computed. The significant ORs (>1) indicated that MPAs manifest more commonly in patients with schizophrenia than in healthy controls.

All analyses were performed using the Comprehensive Meta-Analysis Software package [20].

## Results

### Patients with Schizophrenia vs. Healthy Controls

**Figure 2:**
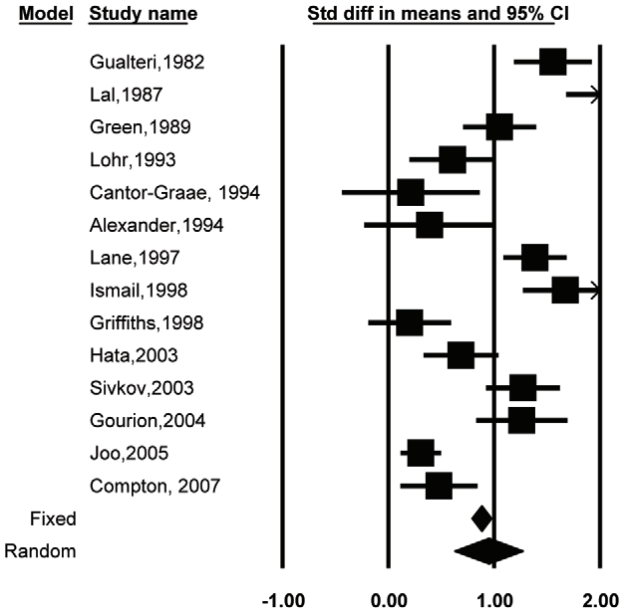
Effect sizes and confidence intervals for each study in the meta-analysis involving patients with schizophrenia and healthy controls.

Fourteen studies [8], [13], [14], [21], [22], [23], [24], [25], [26], [27], [28], [29], [30], [31] compared total MPA scores between patients with schizophrenia and healthy controls, comprising 1207 patients and 1007 controls. Figure 2 shows the individual studies that were included in this analysis. The mean effect sizes for total MPA scores, along with their 95% CIs and homogeneity statistics are shown in Table 1. The difference between patients and controls was in the moderate range (*d* = 0.95, 95% CI = 0.63, 1.27). The *Q* value was significant (*p*<0.001), which implies that results across these studies were heterogeneous. The fail-safe number of studies was 96.

**Table 1:** Results of meta-analyses of MPAs in patients with schizophrenia, relatives of patients, and healthy controls.

	N of studies	N of subjects	std. diff in means	SE	95% CI	Q-value	Fail-safe N
SCZ vs. C	14	1207 SCZ,1007 Controls	0.95	0.16	(0.63, 1.27)	150.56**	96
SCZ vs. R	6	339 SCZ,303 Relatives	0.45	0.08	(0.29, 0.62)	5.04	16
R vs. C	6	303 Relatives,266 Controls	0.32	0.21	(−0.08, 0.73)	32.03**	8

SCZ: Schizophrenia patients.

C: Healthy Controls.

R: Relatives of schizophrenia patients.

**p<0.01.

The meta-regression was performed on account of heterogeneous effect sizes across studies. All the studies included in meta-analysis were used Waldrop Scale or Modified Waldrop Scale. Although the number of the scale items were varied, it did not show significant to interpret the heterogeneous effect size with unrestricted maximum likehood method (*p* = 0.38). We also examined sex ratio among patients, and sex ratio among controls—but none of them were significant.

### Patients with Schizophrenia vs. Unaffected Relatives

**Figure 3:**
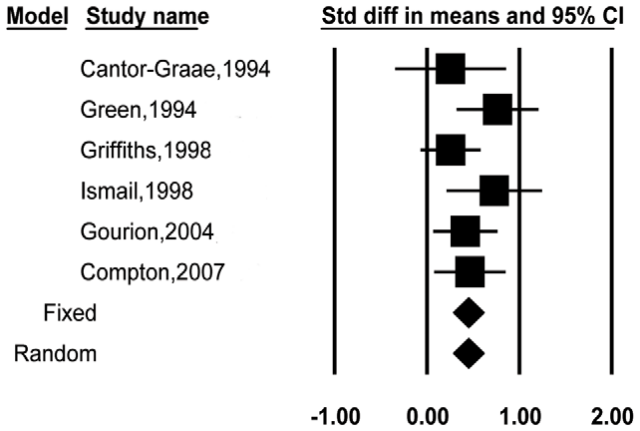
Effect sizes and confidence intervals for each study in the meta-analysis involving patients with schizophrenia and relatives.

Six studies [7], [13], [22], [23], [24], [27] reporting total MPA scores in patients and relatives were included in the second meta-analysis (Figure 3). Table 1 displays the mean effect sizes for these studies, which included 339 patients and 303 relatives of individuals with schizophrenia. The standard difference in means was 0.45 (95% CI = 0.29–0.62). The *Q* value was not significant (*p* = 0.41), indicating that these effect sizes were homogeneous. The fail-safe number of studies was 16.

### Unaffected Relatives vs. Healthy Controls

**Figure 4:**
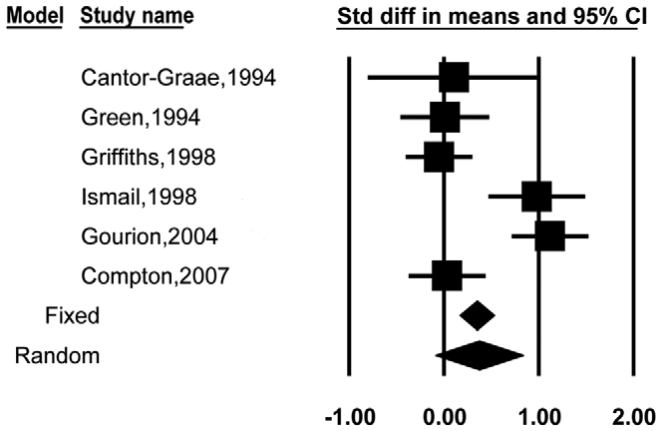
Effect sizes and confidence intervals for each study in the meta-analysis involving unaffected relatives and healthy controls.

Six studies [7], [13], [22], [23], [24], [27] reported total MPAs scores of relatives and controls, which were included in the third meta-analysis. Figure 4 shows the individual studies that were included in this comparison. Table 1 displays the mean effect sizes for 303 relatives and 266 controls. The standard difference in means was 0.32, with a 95% CI = −0.08, 0.73, which included 0. The fail-safe number of studies was 8.

### Individual MPA Items

Ten studies provided the frequency of specific MPA scale items in patients and controls [8], [9], [12], [26], [27], [30], [32], [33], [34], [35]. Table 2 summarizes the pooled results for individual items reported in ≥3 studies. The majority of MPA items were significant, indicating an increased frequency of those items in patients with schizophrenia. The results were homogeneous except for six items (high/steepled palate, furrowed tongue, epicanthus, cleft palate, telecanthus, and big gap between the first and second toes).

**Table 2:** Results of meta-analysis of individual MPA items in patients with schizophrenia and healthy controls.

Item	N	N of patients	N of control	Pooled ORs	95% CI	p-value	Q-value
Tongue with smooth-rough spots	5	272	322	9.86	(2.79, 34.91)	**0.000**	0.84
High/steepled palate	7	695	620	5.12	(3.00, 8.75)	**0.000**	28.26***
Single transverse palmar crease	9	808	815	4.77	(2.47, 9.21)	**0.000**	14.11
Furrowed tongue	9	811	744	4.28	(2.43, 7.55)	**0.000**	17.85*
Syndactyly of 2nd and 3rd toes	5	343	387	4.11	(1.31, 12.87)	**0.015**	4.54
Malformed ears	6	497	467	3.87	(1.80, 8.29)	**0.001**	1.89
Epicanthus	9	866	844	3.74	(2.16, 6.50)	**0.000**	25.51**
Low set ears	8	590	589	2.62	(1.25, 5.53)	**0.011**	4.16
Cleft palate	4	216	224	2.52	(1.13, 5.59)	**0.024**	11.46**
Telecanthus	7	497	467	2.34	(1.30, 4.21)	**0.005**	29.85***
Fine hair	3	183	208	2.30	(0.89, 5.97)	0.086	5.08
3rd toe longer than 2nd	3	136	165	2.25	(0.52, 9.68)	0.278	2.04
Head circumference	5	303	345	2.17	(1.12, 4.23)	**0.022**	1.74
Hair whorls	5	394	438	2.14	(0.97, 4.69)	0.059	5.33
Soft and pliable ears	3	183	208	2.07	(0.66, 6.53)	0.212	2.26
Curved fifth finger	7	439	438	2.04	(1.19, 3.50)	**0.010**	11.33
3rd toe equal to 2nd	4	243	270	2.01	(0.71, 5.69)	0.187	2.70
Asymmetrical ears	6	494	525	1.84	(0.80, 4.26)	0.152	1.88
Big gap between 1st and 2nd toes	6	343	387	1.43	(0.76, 2.68)	0.267	11.38*
Adherent ear lobes	9	808	815	1.26	(0.78, 2.03)	0.343	7.82

*p<0.05,

**p<0.01,

***p<0.001.

## Discussion

In the present study we reviewed the updated literature on MPAs in patients with schizophrenia, unaffected biological relatives, and healthy controls. The combined effect size comparing patients and controls was 0.95, which is consistent with the Weinberg et al. study [5]. We also culled the studies of unaffected relatives of patients with schizophrenia to investigate whether MPAs may represent an endophenotype of schizophrenia. The result of the comparison between patients with schizophrenia and relatives of those with schizophrenia (*d* = 0.45,) indicated a moderate difference between them. On the other hand, the difference between relatives of individuals with schizophrenia and healthy controls (*d* = 0.32) was non-significant. The forest plot (Figure 3) indicated that the results of the studies involving relatives and controls were inconsistent. Therefore, further evidence for MPAs as an endophenotype is necessary, particularly pertaining to the criterion of the marker being present in unaffected family members at a higher rate than in the general population.

Regarding our results on individual MPAs, several studies have investigated whether some items or body region may be more informative than others [4], [5]. Some studies expanded rating scales and assessed previously unmeasured MPAs items [30], [32], [36], such as the use of quantified anthropometric assessments (e.g., facial measurements) in addition to traditional qualitative ratings (e.g., 1 or 2 for presence of the anomaly, 0 for absence); however, the Waldrop scale [6] is currently the most commonly used. Although the number of studies reporting on specific MPAs was limited, a number of individual items had relatively high ORs, and most results were homogeneous. Because most studies did not report on individual items, more research is needed to determine which items are most sensitive for schizophrenia. Schizophrenia is commonly conceptualized as a neurodevelopmental disorder [5], [33]; thus, further studies about individual MPA items would be helpful in better understanding the neurodevelopmental underpinnings of the disorder.

It should be reminded that MPAs are not specific to schizophrenia and are observed in other neurodevelopmental disorders such as autism, learning disabilities, and may be associated with other serious neuropsychiatric disorders such as bipolar disorders and unipolar depression [4], [23], [37]. However, although MPAs are by no means specific to schizophrenia, they appear to be more prevalent among patients with schizophrenia compared to patients with other neuropsychiatric and/or neurodevelpmental disorders.

There are several limitations of the present study, the major one being the small number of studies and limited data reported on unaffected relatives. It should also be noted that the fail-safe number of studies for the comparison between relatives and healthy controls seems quite low. More studies should be conducted in the near future to cross-validate the current findings. In addition, the moderator analysis did not find any significant variable to explain the heterogeneous results. Even so, our meta-analysis result on the difference between patients with schizophrenia and controls was large and reliable. Since there is evidence that MPAs can be a marker for all psychoses, rather than just schizophrenia, it would be interesting to include in the meta-analysis studies that have evaluated also individuals with other types of psychosis. This might also apply to studies that may have evaluated relatives of patients with non-schizophrenia type of psychoses. However, the current meta-analysis does not address this issue owing to the very limited number of available studies. Although the evidence from first-degree relatives was not as robust, it showed a tendency for relatives to have more MPAs than controls. The data suggest that MPAs are a biological marker, and possibly an endophenotype, for schizophrenia.

Taken together, these findings indicate that medium effect size of MPAs have been demonstrated in patients with schizophrenia as compared to healthy controls, and to a lesser extent in unaffected relatives. These findings are consistent with the idea that MPAs may represent a putative endophenotype for schizophrenia. However, more research including first-degree family members is warranted.
